# GABA_A_ Receptor Subunit Transcriptional Regulation, Expression Organization, and Mediated Calmodulin Signaling in Prefrontal Cortex of Rats Showing Testosterone-Mediated Impulsive Behavior

**DOI:** 10.3389/fnins.2020.600099

**Published:** 2020-11-06

**Authors:** Juhee Agrawal, Yogesh Dwivedi

**Affiliations:** Department of Psychiatry and Behavioral Neurobiology, University of Alabama at Birmingham, Birmingham, AL, United States

**Keywords:** impulsivity, testosterone, GABAA receptor, rodent model, transcript level

## Abstract

Testosterone can induce impulsivity, a behavioral impairment associated with various psychiatric illnesses. The molecular mechanisms associated with testosterone-induced impulsivity are unclear. Our earlier studies showed that supraphysiological doses of testosterone to rats induced impulsive behavior, impacted hypothalamic-pituitary-adrenal axis (HPA) and hypothalamic-pituitary-gonadal axis interactions, and altered α_2A_ adrenergic receptors in prefrontal cortex (PFC). Owing to the importance of GABAergic system in impulsivity and memory, the present study examines whether testosterone-mediated impulsivity is associated with changes in the expression of Gamma-Aminobutyric Acid (GABA) A and B receptor subunit transcripts (*Gabra1, Gabra2, Gabra2 transcript variant 2, Gabra3, Gabra4, Gabra5, Gabra6, Gabrb1, Gabrb2, Gabrb3, Gabrg1, Gabrg2, Gabrg3, Gabbr1, Gabbr2*) in rat PFC, and whether testosterone influences GABA_A_ receptor subunit organization. We studied GABA receptor functions by examining GABA receptor-mediated calcium/calmodulin-dependent kinase signaling genes (*Calm1, Calm2, Calm3, Camk2a, Camk2b, Camk2g, Camk2d, Camk4*) in the testosterone-induced impulsivity model. Rats were left untreated as controls (C), gonadectomized (GDX), or GDX and injected with supraphysiological doses of testosterone (T). Impulsive behavior was examined using the go/no-go paradigm. Gene expression was studied using qRT-PCR and GABA_A_ subunit reorganization using cross correlation. Our findings show that expressions of select GABA_A_ receptor subunits (*Gabra3, Gabra5, Gabra6*) were significantly upregulated in PFC of T group compared to GDX or C groups. GABA_A_ receptor subunit organization was different in C, T, and GDX groups. Additionally, *Camk4* expression was significantly downregulated in T compared to C group. Our findings suggest that specific GABA_A_ receptor subunit expression, their reorganization, and *Camk4*-mediated functions may be associated with testosterone-mediated impulsivity.

## Introduction

Impulsivity is described as decision-making or acting without regard to prior thinking. Being a non-unitary construct, impulsivity is often defined as a multidimensional concept related to maladaptive personality traits critical to many neuropsychiatric conditions. Attention-deficit hyperactivity disorder, mood disorders, addiction-related disorders, impulse-control disorders, non-suicidal self-injury, and suicidal behavior are some of the neuropsychiatric conditions that are significantly associated with impulsivity (Bakhshani, 2014; Liu et al., 2017). Despite its significance in neuropsychiatric illnesses, the underlying mechanisms associated with impulsivity are not clearly understood. Recent evidence suggests that increased levels of testosterone and impulsivity are highly correlated in humans (Wu et al., 2019). It has been shown that there is a significant correlation between increased testosterone level and suicide attempts in men (Stefansson et al., 2016). Past studies also suggest an association of increased use of androgen-enhancing drugs, known as anabolic-androgenic steroids, with psychopathologies, such as a blunted hypothalamic-pituitary-adrenal axis (HPA) response and increased risk of suicide attempts (Thiblin et al., 1999; Trenton and Currier, 2005; Melhem et al., 2016). In fact, we have earlier shown that testosterone not only increases impulsivity in rats, but also heightens the interaction of HPA and hypothalamic-pituitary-gonadal axis (HPG) axes in the brains of these rats (Ludwig et al., 2019). In addition, we found that the α_2A_ adrenergic signaling pathway is significantly impacted in the prefrontal cortex (PFC) of rats given supraphysiological doses of testosterone, which was correlated with impulsivity behavior (Agrawal et al., 2019). Our study indicates that neurochemical changes may be central to testosterone-mediated impulsivity.

Gamma*-*Aminobutyric Acid (GABA) is the main inhibitory neurotransmitter in the mammalian central nervous system. In conjunction with excitatory glutamate, GABA is involved in balancing excitatory and inhibitory response, critical in proper brain functioning (Petroff, 2002). GABA binds to GABA receptors to mediate its functions. There are two main types of GABA receptors: (1) GABA_A_ receptor subtypes, which are ligand-gated ion channels and (2) GABA_*B*_ receptor subtype, which are G-protein-coupled receptors (Hayes et al., 2014). GABA_A_ is the primary GABA receptor in the brain, which, due to its chloride ion channel activity, quickly hyperpolarizes the post-synaptic neurons, causing an inhibitory effect. GABA_A_ receptors are pentameric, composed of α, β, γ, δ, and ρ subunits. They have been found to be localized in post-synaptic and extrasynaptic locations (Shrivastava et al., 2011; Hammond, 2015). Based on transcription patterns, GABA_A_ subunits α1 and β2 are co-regulated (Goetz et al., 2007). GABA binds between the α and β subunits, and β subunits regulate ion selectivity of the GABA_A_ receptor (Goetz et al., 2007). The γ2 subunit plays a role in anchoring the receptor to the synapse. Subunit composition determines the GABA_A_ receptors’ conductance and deactivation rate (Goetz et al., 2007). On the other hand, GABA_B_ receptors are composed of GABA_B1_ and GABA_B2_ subunits, forming a heterodimer. GABA_B_ receptors localize pre-synaptically, post-synaptically, and potentially extrasynaptically. GABA_B_ receptors have a slower response than GABA_A_ receptors and play a role in regulating neurotransmission. GABA_B1_ and GABA_B2_ receptor subunits must be co-expressed to render a functional GABA_B_ receptor (Mott, 2015).

Recent studies have shown that GABA may be involved in impulsive behavior. For example, it has been shown that impulsive choice or impulsive action can be associated with expression changes in GABA receptor subunits in rat orbitofrontal cortex (Ucha et al., 2019). Also, GABA level may predict individual differences in rash impulsivity (Boy et al., 2011). A past study examined gonadectomy (GDX) and testosterone treatment on GABA receptors in rats. An alteration in the GABA_A_/benzodiazepine receptors was reported in testosterone-treated animals (Svensson et al., 2000). ^36^Cl^–^ uptake in synaptoneurosomes was increased in the testosterone-treated compared to control rats, suggesting that testosterone may increase GABA_A_ receptor function in this group (Svensson et al., 2003). However, this study did not observe receptor subunit differences between the groups. Furthermore, whether impulsivity induced by testosterone is associated with GABA receptor subunits and their functions has not been studied.

In the present study, we hypothesize that testosterone will disrupt the GABA receptor signaling by increasing their expression in testosterone-treated (T) group compared to control (C) and gonadectomized (GDX) groups. GABA_A_ α, β, and γ subunits were chosen for this study because they comprise the majority of GABA_A_ receptors. Both subunits of GABA_B_ receptors were studied (Shrivastava et al., 2011). We also tested whether testosterone influences GABA_A_ subunit organization by correlating various GABA_A_ receptor subunits in individual groups of rats, as subunit correlations have been found to be disrupted in the brains of subjects with psychiatric disorders such as depression and depressed individuals who died by suicide (Merali et al., 2004). Additionally, we examined the expression of calmodulin (Cal) and calcium/calmodulin-dependent (CaM) protein kinase 2 (CaMk2) as they are considered to be active downstream transducers of GABA receptor functions (Churn and DeLorenzo, 1998; Churn et al., 2002). Furthermore, CaM kinase 4 (CaMk4) gene was studied for its role in GABA receptor expression changes. These studies were performed under the supraphysiological influence of testosterone in PFC in testosterone-induced impulsivity rat model. The PFC was chosen because of its well-studied role in motor control (Robbins and Dalley, 2017; Sholler et al., 2020) decision making (Hiser and Koenigs, 2018; Saberi Moghadam et al., 2019), and emotional processing and regulation (Dixon et al., 2017). Our previous study also indicated that PFC plays a significant role in testosterone-mediated impulsivity (Agrawal et al., 2019).

## Materials and Methods

### Experimental Group Design, Behavioral Testing, and Tissue Collection

#### Rodent Impulsivity Model Preparation

A total of 30 Male Long Evans rats were received from Envigo Laboratories and maintained as described in our previous studies (Agrawal et al., 2019; Ludwig et al., 2019). They were individually housed in a reversed 14 h of light: 10 h of dark photoperiod. They were weighed daily and given food *ad libitum*. The rats were acclimatized for 5 days before starting the experiment. They were divided into a control (C) group and two experimental groups, both of which received gonadectomies to eliminate testosterone. The age of rats was ∼6 weeks at the time of gonadectomy. One of the experimental groups was given daily injections of excessive testosterone and is known as the testosterone (T) group. The group with the gonadectomy only is known as the gonadectomized (GDX) group. Experimental procedures were approved by the IACUC of the University of Alabama at Birmingham and all procedures were conducted in strict adherence to the National Institutes of Health (NIH) Guide for the Care and Use of Laboratory Animals.

Rats receiving a GDX or testosterone injections were subjected to the same protocol used previously in this lab (Agrawal et al., 2019; Ludwig et al., 2019). To summarize, rats received isoflurane as an anesthetic (5% induction, 1–3% maintenance), and their vitals were checked regularly to minimize the risk of cardiorespiratory failure. Rats also received carprofen (5 mg/kg, subcutaneous) and buprenorphine (0.1 mg/kg, subcutaneous) before incision. For the incision, the aseptic technique was followed. Rats’ abdomen were shaved, and betadine was used to cleanse. Then, a single transverse incision in the caudal abdomen was made, and using blunt forceps, the testicular fat pad on one side was pulled through the incision. Both testes and epididymis were removed using a hemostat and ligature for control. Monocryl sutures were used to close the incisions. During and after surgery, rats’ body temperatures were monitored using a heating pad. 24 h post-surgery, rats received a second subcutaneous injection of carprofen (5 mg/kg).

**FIGURE 1:**
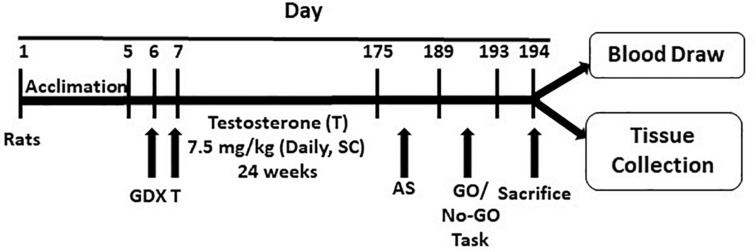
Schematic diagram showing the experimental protocol. The protocol is detailed in the text. GDX, gonadectomy; T, testosterone; AS, autoshaping; SC, subcutaneous.

A schematic diagram showing the experimental protocol is mentioned in Figure 1. One day after surgery, GDX rats were randomly assigned to receive testosterone injections and form the testosterone (T) group. They were subcutaneously injected with testosterone propionate (7.5 mg/kg, dissolved in 0.1 mL corn oil). This dose is equivalent to a heavy steroid dose in humans, and has been used in previous studies (Wood et al., 2013; Cooper et al., 2014). Injections were given daily for a 24-week period. GDX rats, not receiving testosterone, received corn oil injections of equal volume and for the same duration. Rats were weighed every 2 weeks, and the daily dose of testosterone was adjusted for their weight.

#### Behavioral Testing

After 24 weeks of testosterone administration, rats were subjected to the go/no-go task paradigm to test their impulsive behavior. This paradigm is described in our previous study (Agrawal et al., 2019). Rats placed in sound-attenuated and ventilated chambers were autoshaped for 15 days to learn that pressing the lever in the chamber would result in the dispensing of 45 mg sucrose pellets. Rats who successfully learned (defined as pressing the lever at least 20 times during 30 min of the “go” phase, when the light was on) continued testing, and those who did not were removed from testing.

Rats that continued testing moved to the “go/no go” task phase of the experiment. Rats were to press the lever to dispense sucrose pellets during the “go” phase, which was signaled by a bright white light and continued with a house light. They were not to press the lever during the “no-go” phase, which was signaled by a red light and continued with no light. Each phase was 10 min, with 10 s timeouts between phases. In total, there were 4 “go” and 4 “no-go” phases which alternated for a total of an 80 m session. Rats underwent 3 sessions that were included in results, and they completed one warm-up session of this paradigm that was not included in results. The sessions were controlled by Med-PC IV^®^ software ran on a computer. Impulsive behavior was defined as pressing the lever during the “no-go” phase. Impulsive behavior was indexed by the go/no-go ratio, which was the number of times the lever was pressed during the “go” phase compared to the number of times it was pressed during the “no-go” phase. A high ratio indicated low impulsive behavior, while a low ratio indicated high impulsive behavior. If variance in this ratio between the 3 sessions was <10% for any individual rat, results were recorded. Individual responses were averaged based on experimental group (C, T, and GDX).

Twenty-four hours after the last session, rats were decapitated, trunk blood was collected, and brains were flash frozen and stored at −80°C. The PFC was dissected out from 300 μm sections prepared from the frozen brain on a cryostat (Leica CM1950, Leica, Germany) and PFC was carved out precisely by scalpel using the Rat Brain Atlas and stored at −80°C until further analyses.

#### Testosterone Levels

Serum isolated from trunk blood was tested for testosterone levels using an ELISA kit (Abcam, MA, United States). One-way ANOVA and Student’s *t*-tests were performed between groups to determine variance and statistical significance. Data are presented as testosterone concentration values in ng/mL.

### Expression Levels of GABA Receptor Subunits

#### Primer Design

Primers were designed using Rat Genomic Database (RGD) and NCBI BLAST search tool. Primer sequences were designed to target the 3′ end of the gene and accounted for all the transcript variants for each gene. Primers were designed to have low self-complementarity, low self-3′ complementarity, low GC%, and melt temperatures within 2°C of each other. A list of all primers used in the gene expression assay can be found in Table 1.

**TABLE 1:** Rat-specific primers for amplifying mRNA transcripts.

Genes	Forward (5′–3′)	Reverse (5′–3′)
*Gabra1*	TGC CTG TGT TTC CCT AAA ACG	AGG CAG GAC CAA ATC AAA CAA T
*Gabra2*	CCT CTG GCC TGG TTG CTT TA	GCT CTC TCC TTA TGT GTG TCA AG
*Gabra2-transcript variant 2*	ATC CTT CTG TCC CAC CCT TTT AG	CAT CAA CAT AAT CCC CAG CAC T
*Gabra3*	GGC ATG ATC CGC AAA CAG TAG	CTC TGG GGT TTG GGA TTT GGA
*Gabra4*	CCT TCT GGA TCT GGC ACA AGT	AAT GCC CCA AAT GTG ACT GG
*Gabra5*	CCA TTT TTC CCA GCC AAC AGA	TGT ACC CGA GGA TCT TTG CTT T
*Gabra6*	AGC TGT ATG CTT TGC GTT TGT	CTT TCG GCT TTC TGG GAC TG
*Gabrb1*	TCC TTT CCT CCT CGC TTG TTT	AAC TGG AAG GCG GAA TCT CTT
*Gabrb2*	TGT CAA CAA GAT GGA CCC ACA	ATG CTG GAG GCA TCA TAG GC
*Gabrb3*	TAC GCA TAC ATA CCA TAC ATT TTG C	TGT GTT TCT CGC CCT CAT TCT
*Gabrg1*	AGA CTT GGT TCG ATA GCC GTT	GGC GAT TGG GCG TTG TTA TC
*Gabrg2*	TCA CAG CAA TGG ATC TCT TCG T	TGG TAG GGG CAG GGT TTT TC
*Gabrg3*	TCG AAA GCC AAC CAT CAG GAA	GTG GGG GTC TCA TAT CCA GG
*Gabbr1*	CTG CGT CGT GGT TTC CTT TC	TCC GTG CCT TCA TTT GGT CA
*Gabbr2*	AAC AAG CCC CAC AAA GTG ATT	TCT AGG AAA TGG ACC GTG TGT T
Calm1	CTC TGA TGG GG GAC CAA CTC	CGC AGT TAG AGA TGA AAG GCT G
Calm2	TGG AGT TGG TCA AAT GAG GGA	TGT CCA TAG TCC ACG CAG AG
Calm3	CAA TGT GGG CAG TTC AGT CG	GTC CCA GGA AAA GCC ACT TG
Camk2a	CAG CCG ATG AAG GAG CAA AC	AGG AGT CCA GCC AGT GAC TAT
Camk2b	CCC CTA CAA ATC AAG CCA AGG	ACG GAA GAT GGT GTC CAC TC
Camk2d	CTC TTG TTT TGC TGT TGG GCT	TGC TGA GAC ATT TGA GTC CGA
Camk2g	GCT GGT GCT TGG ATT TAG CC	AAG CCA GCA AAA CGA AAC CC
Camk4	ACA GAT GCA AAC AGA AGG GGA	TTG GAT GTG AGA GGC GAA GAA

#### RNA Isolation and qPCR-Based Gene Expression Analysis of GABA Receptor Subunits and Signaling Genes in Rat PFC

RNA was isolated following TRIzol^®^ (Life Technologies, United States) method as described earlier (Roy et al., 2017). RNA purity was determined by measuring the optical density with an absorbance ratio of 260/280 (NanoDrop 2000c, Thermo-Scientific, Waltham, MA, United States), and integrity was tested following denaturing agarose gel electrophoresis. All samples had 260/280 ratio >1.8 and demonstrated 28S:18S rRNA ratio of 2:1 on agarose gel.

The single-stranded cDNA was prepared following the previously described method (Timberlake et al., 2018). Following the preparation of mRNA specific first strand cDNA, relative transcript abundance of coding genes was measured with quantitative real-time PCR (Stratagene MxPro3005, La Jolla, CA, United States) method. With the help of EvaGreen chemistry (Applied Biological Material Inc., Canada), qPCR amplification for the specific gene was performed using gene-specific forward and reverse primers as mentioned in Table 1. Primers were designed for the following genes: GABA_A_ receptor subunits α1–6 (*Gabra1*, *Gabra2, Gabra2-transcript variant 2, Gabra3, Gabra4, Gabra5, Gabra6*), GABA_A_ receptor subunits β1–3 (*Gabrb1, Gabrb2, Gabrb3*), GABA_A_ receptor subunits γ1–3 (*Gabrg1, Gabrg2, Gabrg3*), GABA_B_ receptor subunits 1–2 (*Gabbr1, Gabbr2*), calmodulin genes 1–3 (*Calm1, Calm2, Calm3*), calcium/calmodulin-dependent protein kinase type II subunits α, β, γ, and δ (*Camk2a, Camk2b, Camk2g, Camk2d*), and calcium/calmodulin-dependent protein kinase type IV (*Camk4*). The possibility of primer dimer formation and secondary product amplification was ruled out by running template-free samples. *Gapdh* normalized gene expression values were used to determine the relative gene expression levels of individual transcripts following Livak’s ΔΔCt calculation method (Livak and Schmittgen, 2001).

### GABA_A_ Receptor Subunit Transcript Reorganization

GABA_A_ receptor subunit mRNA reorganization was determined by correlating expression levels of various GABA_A_ receptor subunits (*Gabra1*, *Gabra2, Gabra2-transcript variant 2, Gabra3, Gabra4, Gabra5, Gabra6*, *Gabrb1, Gabrb2, Gabrb3*, *Gabrg1, Gabrg2*, and *Gabrg3*) in C, T, and GDX groups independently using Pearson Product Moment Analysis.

## Results

### Serum Testosterone Levels

As can be seen in our prior study (Agrawal et al., 2019), average testosterone concentration for the testosterone (T) group (*n* = 5) was 11.6 ng/mL (SEM = 4.67), compared to the average concentration for the control (C) group (*n* = 6) of 1.63 ng/mL (SEM = 0.4), and the average concentration for the gonadectomized (GDX) group (*n* = 6) of 0 ng/mL (SEM = 0.0061). T group concentration was significantly higher than both C group (*p* = 0.04) and GDX group (*p* = 0.02). GDX group concentration was significantly lower than C group (*p* = 0.001).

### Impulsive Behavior Testing Results

As detailed in our previous study, T group rats had significantly lower go/no-go ratios than those in the GDX and C groups (Agrawal et al., 2019). T group (*n* = 5) had a ratio of 0.54 (SEM = 0.035), C group (*n* = 6) had a ratio of 0.87 (SEM = 0.073), and GDX group (*n* = 6) had a ratio of 0.76 (SEM = 0.075). T group’s ratio was significantly lower than the ratio for C group (*p* = 0.004) and GDX group (*p* = 0.03). There was no significant difference between the ratios of C group and GDX group (*p* = 0.32).

### GABA Receptor Gene Expression Analysis

Gene expression analysis was done in rat PFC to determine the effect of a supraphysiological dose of testosterone (T) on GABA receptor subunit genes and genes associated with calcium-dependent calmodulin kinase signaling pathway compared to rats with normal levels of testosterone (C) and rats with no testosterone due to gonadectomy (GDX).

**FIGURE 2:**
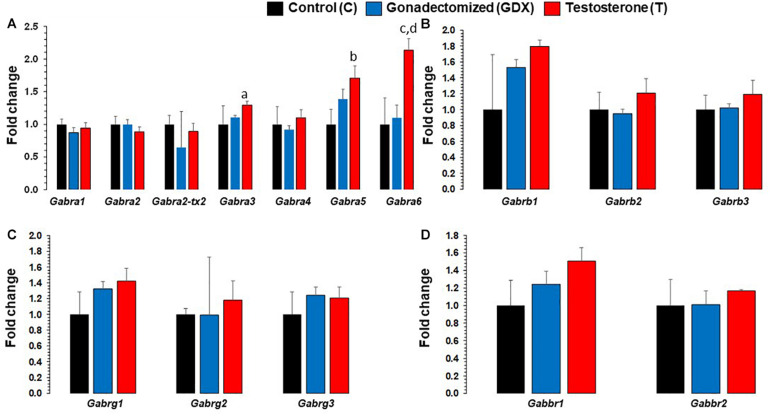
Bar diagram showing gene expression results for GABA_A_ and GABA_B_ receptor subunits in prefrontal cortex across control (C, *n* = 4), gonadectomized (GDX, *n* = 5), and testosterone (T, *n* = 4) groups. Data are the mean ± SEM. **(A)** Expression changes in GABA_A_ α receptor subunits across T and GDX groups. The T rats displayed significantly upregulated *Gabra3* mRNA transcript levels (^a^*p* = 0.0087) compared with the GDX group. Similar significant expression upregulation was seen for *Gabra6* gene in T rats as compared to GDX rats (^d^p = 0.009). The *Gabra5* (^b^*p* = 0.037) and *Gabra6* (^c^*p* =0.047) were all found to be upregulated in the T group compared to the C group. No significant changes in *Gabra1, Gabra2, Gabra2-tx2, Gabra4* were noticed when comparing T group of rats with GDX rats, nor when comparing T rats with C rats. **(B)** Expression levels of GABA_A_ β receptor subunits across C, T, and GDX groups. No significant changes in *Gabrb1, Gabrb2, and Gabrb3* were noted when comparing T group of rats with GDX rats, or T rats with C rats. **(C)** Expression levels of GABA_A_ γ receptor subunits across C, T, and GDX groups. No significant differences in *Gabrg1, Gabrg2, and Gabrg3* were noted when comparing T group of rats with GDX rats, or T rats with C rats. **(D)** Expression levels of GABA_B_ receptor subunits across C, T, and GDX groups. No significant changes in *Gabbr1 and Gabbr2* were noted when comparing T group of rats with GDX rats, or T rats with C rats.

Expression level changes of all GABA_A_ and GABA_B_ receptor subunit transcripts in PFC are presented in Figure 2. Fold changes between groups were standardized with C group as the reference (fold change = 1). *Gabra3* fold change was 1.29 (SEM = 0.058) for T group and 1.10 (SEM = 0.035) for GDX group, compared to 1 (SEM = 0.28) for C group. T group’s *Gabra3* expression was significantly upregulated compared to GDX (*p* = 0.008), but not significant compared to C group (*p* = 0.244). *Gabra5* fold change was 1.71 (SEM = 0.18) for T group and 1.39 (SEM = 0.15) for GDX group, compared to 1 (SEM = 0.22) for C group. T group’s *Gabra5* expression was significantly upregulated compared to C (*p* = 0.038), but not significant compared to GDX group (*p* = 0.24). *Gabra6* fold change was 2.14 (SEM = 0.17) for T group and 1.10 (SEM = 0.19) for GDX group, compared to 1 (SEM = 0.40) for C group. T group’s *Gabra6* expression was significantly upregulated compared to both GDX (*p* = 0.009) and C (*p* = 0.047).

Other GABA_A_ receptor subunits (*Gabra1, Gabra2, Gabra2-transcript variant 2, Gabra4, Gabrb1, Gabrb2, Gabrb3, Gabrg1, Gabrg2*, and *Gabrg3*) were not significantly altered between groups. Neither of the GABA_B_ receptor subunits (*Gabbr1 and Gabbr2*) were significantly different between groups.

### Calmodulin and CaM Kinase Gene Expression Analysis

**FIGURE 3:**
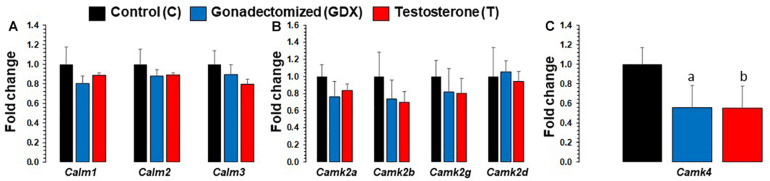
Bar diagram showing gene expression results for calmodulin and Calcium/calmodulin-dependent protein kinase subunits in prefrontal cortex across control (C, *n* = 4), gonadectomized (GDX, *n* = 5), and testosterone (T, *n* = 4) groups. Data are the mean ± SEM. **(A)** Expression levels of *Calm* genes across C, T, and GDX groups. No significant differences were noted in *Calm1 and Calm2 genes, however, calm3* was nearly significant (*p* = 0.06) when comparing T rats to C rats. **(B)** Expression levels of *Camk2* genes across C, T, and GDX groups. No significant differences were found in the following genes: *Camk2a, Camk2b, Camk2g, and Camk2d* when comparing T to GDX, T to C, or GDX to C. **(C)** Expression changes in *Camk4* across T and GDX groups. One-way ANOVA found significant differences across groups in the *Camk4* gene. The T rats displayed significantly downregulated *Camk4* mRNA transcript levels (^b^*p* = 0.02) compared with the C group. Similar significant expression downregulation was seen for *Camk4* in GDX rats as compared to C rats (^a^*p* = 0.03).

mRNA expression changes of all calmodulin and CaMK subunit genes in PFC are presented in Figure 3. Based on ANOVA, significant expression differences were found across groups *in Camk4*. *Camk4* fold change was 0.55 (SEM = 0.222) for T group and 0.55 (SEM = 0.227) for GDX group, compared to 1 (SEM = 0.173) for C group. *Camk4* expression was significantly downregulated in T group compared to C group (*p* = 0.02). *Camk4* expression was also significantly downregulated in GDX group compared to C group (*p* = 0.03). No significant differences were found in *Camk4* expression between T and GDX groups. Additionally, *Calm3* expression was nearly significantly downregulated in T group compared to C group (*p* = 0.069), but not to GDX (*p* = 0.233). No significant differences were found in the expression levels of *Calm1, Calm2, Camk2a, Camk2b, Camk2g*, and *Camk2d.*

### GABA_A_ Receptor Subunit Transcript Organization

**FIGURE 4:**
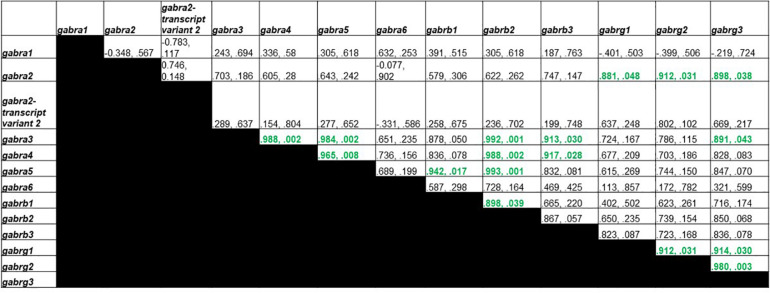
Pearson Correlations for C group GABA_A_ receptor subunit gene expression. Data are presented as R and p. R is the correlation coefficient, and p is the *p*-value from student’s *t*-test. 17 significant correlations were found between *Gabra3* and *Gabra4*, *Gabra3* and *Gabra5*, *Gabra4* and *Gabra5*, *Gabra5* and *Gabrb1*, *Gabra3* and *Gabrb2*, *Gabra4* and *Gabrb2*, *Gabra5* and *Gabrb2*, *Gabrb1* and *Gabrb2*, *Gabra3* and *Gabrb3*, *Gabra4* and *Gabrb3*, *Gabra2* and *Gabrg1*, *Gabra2* and *Gabrg2*, *Gabrg1* and *Gabrg2*, *Gabra2* and *Gabrg3*, *Gabra3* and *Gabrg3*, *Gabrg1* and *Gabrg3*, and *Gabrg2* and *Gabrg3*.

Figure 4 shows Pearson correlations for GABA_A_ receptor subunit gene expression in the control group. Significant correlations were found in 17 different subunit combinations: *Gabra3* and *Gabra4*, *Gabra3* and *Gabra5*, *Gabra4* and *Gabra5*, *Gabra5* and *Gabrb1*, *Gabra3* and *Gabrb2*, *Gabra4* and *Gabrb2*, *Gabra5* and *Gabrb2*, *Gabrb1* and *Gabrb2*, *Gabra3* and *Gabrb3*, *Gabra4* and *Gabrb3*, *Gabra2* and *Gabrg1*, *Gabra2* and *Gabrg2*, *Gabrg1* and *Gabrg2*, *Gabra2* and *Gabrg3*, *Gabra3* and *Gabrg3*, *Gabrg1* and *Gabrg3*, and *Gabrg2* and *Gabrg3*.

**FIGURE 5:**
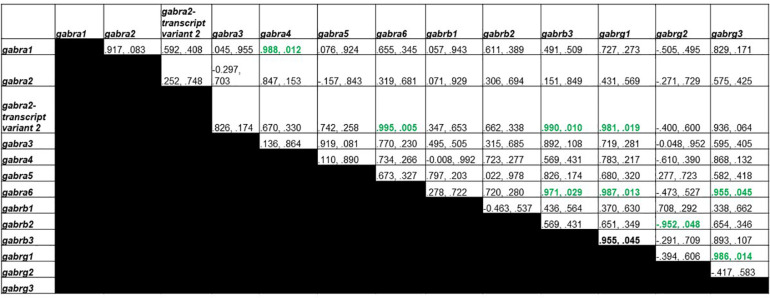
Pearson Correlations for T group GABA_A_ receptor subunit gene expression. Data are presented as R and p. R is the correlation coefficient, and p is the *p*-value from student’s *t*-test. 9 significant correlations were found between *Gabra1* and *Gabra4*, *Gabra2-transcript variant 2* and *Gabra6*, *Gabra2-transcript variant 2* and *Gabrb3*, *Gabra6* and *Gabrb3*, *Gabra2-transcript variant 2* and *Gabrg1*, *Gabra6* and *Gabrg1, Gabrb2* and *Gabrg2, Gabra6* and *Gabrg3*, and *Gabrg1* and *Gabrg3*.

Figure 5 shows Pearson correlations for the T group GABA_A_ receptor subunit gene expression. Significant correlations were found in 9 different subunit combinations: *Gabra1* and *Gabra4*, *Gabra2-transcript variant 2* and *Gabra6*, *Gabra2-transcript variant 2* and *Gabrb3*, *Gabra6* and *Gabrb3*, *Gabra2-transcript variant 2* and *Gabrg1*, *Gabra6* and *Gabrg1, Gabrb2* and *Gabrg2, Gabra6* and *Gabrg3*, and *Gabrg1* and *Gabrg3*.

**FIGURE 6:**
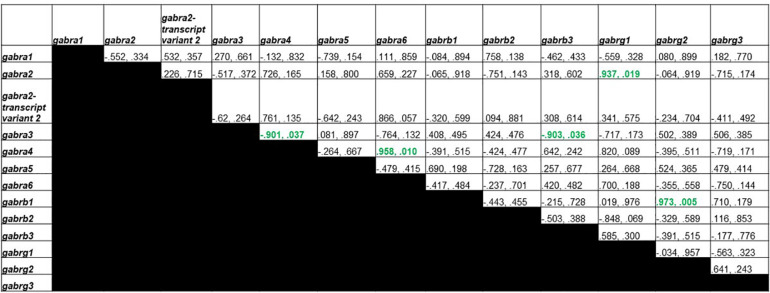
Pearson Correlations for GDX group GABA_A_ receptor subunit gene expression. Data are presented as R and p. R is the correlation coefficient, and p is the *p*-value from student’s *t*-test. 5 significant correlations were found between *Gabra3* and *Gabra4*, *Gabra4* and *Gabra6*, *Gabra3* and *Gabrb3*, *Gabra2* and *Gabrg1*, and *Gabrb1* and *Gabrg2.*

Figure 6 shows Pearson correlations for GDX group GABA_A_ receptor subunit gene expression. Significant correlations were found in 5 different subunit combinations: *Gabra3* and *Gabra4*, *Gabra4* and *Gabra6*, *Gabra3* and *Gabrb3*, *Gabra2* and *Gabrg1*, and *Gabrb1* and *Gabrg2*.

## Discussion

In this study, we analyzed the effects of testosterone-induced impulsivity on GABA_A_ and GABA_B_ receptor subunit mRNA levels in rat PFC. As indicated in the results section, we found that only GABA_A_, but not GABA_B_ receptor transcript levels, showed changes. Within GABA_A_ receptors, testosterone had a subunit-specific effect on *Gabra3, Gabra5*, and *Gabra6* transcript levels. We predict that changes in only GABA_A_ but not in GABA_B_ could be due to the underlying differences in receptor composition between these two receptors and their distinct mechanisms of interaction with specific ligands and consequent downstream functions (Hammond, 2015; Mott, 2015). GABA_B_ receptors are inhibitory G-protein coupled receptors and are found in both pre- and post-synaptic locations (Wu and Sun, 2015). GABA_A_ receptors are ionotropic, fast hyperpolarizing, and expressed throughout the brain. Of various GABA_A_ receptor subunits, GABA_A_ 4, 5, and 6 subunits are extrasynaptic (Wu and Sun, 2015). These receptors have a high GABA binding affinity and are persistently activated even at a low concentration of GABA, resulting in tonic inhibition (Farrant and Nusser, 2005). This tonic inhibition could be a compensatory mechanism shown in testosterone-induced impulsivity. Because testosterone is a hydrophobic steroid hormone, it is able to cross the Blood-Brain Barrier and target GABA receptors This effect could be localized to several brain regions by signaling molecules directing testosterone’s activity, or by increased binding affinity for location-specific receptors. Testosterone may target GABA receptors that are not present at the synapse itself but are still along the neuronal cell surface. Increased GABA_A_ receptor activity in impulsivity is consistent with previous findings in rodent models. For example, a study found that increased GABA_A_ receptor activity in the infralimbic PFC, rather than the prelimbic PFC, increased impulsive responding in rats (Murphy et al., 2012). Another study examined GABA receptors in gambling disorder and found that individuals with gambling disorder had higher levels of GABA_A_ receptors in the right hippocampus compared to healthy controls (Mick et al., 2017). While this study focused on testosterone’s effect on the PFC due to the PFC’s well-studied role in impulsivity and our previous findings showing a PFC-specific alteration in noradrenergic signaling, it is important to note that other brain regions may also be involved in impulsivity. For instance, a study recently found a neural circuit connecting the ventral hippocampus to the PFC in food impulsivity inhibition (Hsu et al., 2018). Because our previous study indicated the localized effect that testosterone had on the PFC and not on the hippocampus or amygdala, we chose to continue focusing on the PFC in this study for testosterone-mediated alterations of other neurotransmitter receptor signaling pathways (Agrawal et al., 2019).

We observed that increased testosterone level was associated with altered expression of specific GABA_A_ subunit mRNA expression. The reasons for the altered expression of specific subunit genes of GABA_A_ receptors are not clear at the present time; however, several transcription factors have been found to be associated with promoter regions of these genes (Steiger and Russek, 2004). These include early growth response factors 1 and 3, Myc associated growth factor, and zinc binding protein 9 and 89. Whether these transcription factors influence these subunits needs to be further studied. In a recent study, we observed that testosterone interacts with the HPA axis through molecules that influence stress response (Ludwig et al., 2019). For example, corticotropin-releasing hormone (Crh) and FK506 binding protein 5 (Fkbp5) genes were significantly upregulated in GDX rats. Interestingly, there is a significant interaction between CRH and GABA receptor systems. It has been reported that pharmacological agents that influence GABA can profoundly impact CRH systems (Cullinan and Wolfe, 2000; Skelton et al., 2000; Stout et al., 2001; Gilmor et al., 2003). Also, CRH is uniquely expressed in glutamic acid decarboxylase (GAD)-positive neurons (Cullinan and Wolfe, 2000). GAD is an enzyme responsible for the conversion of glutamate to GABA. Several GABA_A_ receptor subunits are altered within CRH neurons (Cullinan and Wolfe, 2000). Thus, the possibility of HPA axis-responsive genes and changes in specific subunits of GABA by testosterone cannot be ruled out. Whereas an association between CRH-GABA has been established under stressful conditions (Yan et al., 1998), whether this interaction plays a role in impulsivity needs to be tested.

Our study also assessed cross-correlations between GABA_A_ receptor subunit mRNA levels in control (C), testosterone (T), and gonadectomized (GDX) groups. We found that while C group rats had 17 significant correlations, T rats had 9, and GDX had only 5. These differences in subunit composition indicate a loss of co-regulated genes in GDX group and testosterone partially restored this loss. Interestingly, studies have examined the organization of various GABA receptor subunits and found that in certain psychiatric conditions, such as depression and suicide, not only are mRNA expression altered in specific GABA receptor subunits, but inter-relations between various subunits differ (Brambilla et al., 2003; Merali et al., 2004; Poulter et al., 2010; Yin et al., 2016). Although these studies did not correlate GABA subunit organization with impulsivity, it is quite possible that altered stoichiometric organization could influence neuronal firing patterns and their timing, which may be consequential in impulsivity.

Protein phosphorylation is one of the main mechanisms of regulating GABA receptor functions. It has been shown that CamKII-dependent phosphorylation can increase GABA receptor binding, and thus modulate GABA receptor-mediated chloride ion channel activity (Churn and DeLorenzo, 1998). It has also been reported that CaMKII activation can lead to an increase in specific GABA receptor subunits (Churn et al., 2002). Calmodulin genes *Calm1*, *Calm2*, and *Calm3* have been studied to produce identical calmodulin proteins. Calmodulin is a calcium-binding protein that plays a role in memory formation. The calcium/calmodulin dependent kinase IIa (*Camk2a)* has been found to play a role in integrating Ca^2+^ signals in dendritic spines (Chang et al., 2019), and increasing expression after long-term potentiation (Thomas et al., 1994). *Camk2b* is found to be more prevalent in sympathetic neurons (Ma et al., 2015), and to play a role in tethering the *Camk2* protein complex to dendritic spines (Shen et al., 1998). While *Camk2a* and *Camk2b* are primarily found in the nervous system, *Camk2g* and *Camk2d* are found throughout the body (Gray and Heller Brown, 2014). *Camk2g* functions to transport calmodulin from the cell surface to the nucleus (Malik et al., 2014). *Camk2d* is predominantly found in cardiac tissue, and its expression alters during cardiomyocyte differentiation, heart failure, and ischemia (Gray and Heller Brown, 2014). Also, both calmodulin and a *Camk2* inhibitor can block the potentiating effect of Ca^2+^ on Cl^–^ current gated by GABA_A_ receptors (Aguayo et al., 1998). Moreover, glutamatergic synaptic activity is controlled by GABA_A_ receptors by inhibiting glutamate release via Ca^2+^/calmodulin-dependent signaling (Long et al., 2009). Although the relationship of GABA_A_ receptors and CaMKIV is not well established, a histochemical study shows CaMKIV is expressed in a subgroup of GABAergic neurons in all layers of cortical interneurons of adult monkey area V1 in which parvalbumin was present (Lalonde et al., 2004). In this study, we examined the expression of calmodulin genes and various CaM kinases: *Calm1, Calm2, Calm3, Camk2a, Camk2b, Camk2g, Camk2d, and Camk4.* It was observed that *Camk4* gene expression was significantly lower when comparing T rats to C rats and GDX rats to C rats. Additionally, *Calm3* was lower when comparing T rats to C rats, but it could not reach significance level. It appears that *Camk4* might be involved in differential regulation of *Gabra3, Gabra5*, and *Gabra6* receptors. However, changes in *Camk4* are rather surprising given that this kinase is not very well linked to GABA_A_ receptor functioning (Houston et al., 2009). The possibility of GABA-independent functions of *Camk4* cannot be ruled out.

## Conclusion and Limitations

Altogether, this is the first GABA_A_ and GABA_B_ subunit gene expression analysis in an animal model of testosterone-induced impulsivity. We show that the impulsivity response may be GABA_A_ subunit-specific when involving supraphysiological concentrations of testosterone. Also, the organization of GABA_A_ receptor subunits is quite different between control, testosterone, and GDX rats. Further, this study shows that subunit-specific effects of testosterone may be associated with calmodulin/calcium-dependent kinases. Our study thus provides an interplay between testosterone, impulsivity and GABAergic functions. From a clinical perspective, GABA_A_ receptors are more frequently used as therapeutic targets when treating disorders such as anxiety and epilepsy (Arin et al., 2018). In the future, it will be interesting to translate this study to a human level to examine if GABA receptor functions are altered during impulsivity, particularly in suicidal people where this interaction has been shown to play a critical role in attempted and completed suicide (Sher et al., 2017; Lenz et al., 2019). There were a few limitations to our study. This study did not have a group with a normal amount of testosterone (created by GDX + normal amount of testosterone). However, in the results section, testosterone level analyses from serum confirmed the following 3 groups: a testosterone-depleted GDX group, a group with supraphysiological levels of testosterone (T+) and another group with physiological levels of testosterone (C). Thus, the C rats were used for normal level of testosterone. One caveat of the study is that the results were derived from the transcript (mRNA) levels of GABA_A_ receptor subunits and CaM kinases and not from protein levels. Thus, there is a possibility that changes in transcription may not necessarily reflect changes in protein. Similarly, CaM kinase activity was not studied. However, as mentioned above, based on mRNA expression levels, Poulter et al. (2010) showed that GABA_A_ receptor organization was altered in the brain of depressed subjects. These investigators convincingly argued that the relative mRNA abundance of GABA_A_ receptor subunits would be a mechanism that ensured proportional abundance of protein. In addition, Brooks-Kayal et al. (1999) demonstrated that subunit mRNA levels correlated closely with receptor pharmacology within individual dentate granule cells, which could be similar to those predicted by studies of recombinant receptors. Nevertheless, our present study needs to be confirmed at the protein level to reach a definite conclusion. In addition, though the most common GABA_A_ receptors were chosen to study, other subunits such as δ and ρ could also be studied in the future.

## Data Availability Statement

The raw data supporting the conclusions of this article will be made available by the authors, without undue reservation.

## Ethics Statement

The experimental procedures were approved by the IACUC of the University of Alabama at Birmingham.

## Author Contributions

JA performed the experiments and analyzed the data. YD conceptualized the idea. JA and YD co-wrote the manuscript. Both authors contributed to the article and approved the submitted version.

## Conflict of Interest

The authors declare that the research was conducted in the absence of any commercial or financial relationships that could be construed as a potential conflict of interest.

## References

[B1] Agrawal J. Ludwig B. Roy B. Dwivedi Y. (2019). Chronic testosterone increases impulsivity and influences the transcriptional activity of the Alpha-2A adrenergic receptor signaling pathway in rat brain. *Mol. Neurobiol.* 56 4061–4071. 10.1007/s12035-018-1350-z 30264294 PMC6502699

[B2] Aguayo L. G. Espinoza F. Kunos G. Satin L. S. (1998). Effects of intracellular calcium on GABAA receptors in mouse cortical neurons. *Pflügers Archiv.* 435 382–387. 10.1007/s004240050527 9426294

[B3] Arin B. Martin W. Anne Kerstin L. Richard W. O. (2018). *GABAA Receptor Physiology and Pharmacology.* Oxford: Oxford University Press.

[B4] Bakhshani N.-M. (2014). Impulsivity: a predisposition toward risky behaviors. *Int. J. High Risk Behav. Addict.* 3:e20428. 10.5812/ijhrba.20428 25032165 PMC4080475

[B5] Boy F. Evans C. J. Edden R. A. Lawrence A. D. Singh K. D. Husain M. (2011). Dorsolateral prefrontal gamma-aminobutyric acid in men predicts individual differences in rash impulsivity. *Biol. Psychiatry* 70 866–872. 10.1016/j.biopsych.2011.05.030 21757187 PMC3192031

[B6] Brambilla P. Perez J. Barale F. Schettini G. Soares J. C. (2003). GABAergic dysfunction in mood disorders. *Mol. Psychiatry* 8 721–737. 10.1038/sj.mp.4001362 12888801

[B7] Brooks-Kayal A. R. Schumate M. D. Jin H. Lin D. D. Rikhter T. Y. Hollway K. L. (1999). Human neuronal γ-aminobutyric acidA receptors: coordinated subunit mRNA expression and functional correlates in individual dentate granule cells. *J. Neurosci.* 19 8312–8318. 10.1523/JNEUROSCI.19-19-08312 10493732 PMC6783026

[B8] Chang J.-Y. Nakahata Y. Hayano Y. Yasuda R. (2019). Mechanisms of Ca2+/calmodulin-dependent kinase II activation in single dendritic spines. *Nat. Commun.* 10:2784. 10.1038/s41467-019-10694-z 31239443 PMC6592955

[B9] Churn S. B. DeLorenzo R. J. (1998). Modulation of GABAergic receptor binding by activation of calcium and calmodulin-dependent kinase II membrane phosphorylation. *Brain Res.* 809 68–76. 10.1016/S0006-8993(98)00834-8 9795142

[B10] Churn S. B. Rana A. Lee K. Parsons J. T. De Blas A. Delorenzo R. J. (2002). Calcium/calmodulin-dependent kinase II phosphorylation of the GABAA receptor α1 subunit modulates benzodiazepine binding. *J. Neurochem.* 82 1065–1076. 10.1046/j.1471-4159.2002.01032.x 12358754

[B11] Cooper S. E. Goings S. P. Kim J. Y. Wood R. I. (2014). Testosterone enhances risk tolerance without altering motor impulsivity in male rats. *Psychoneuroendocrinology* 40 201–212. 10.1016/j.psyneuen.2013.11.017 24485492 PMC3919461

[B12] Cullinan W. E. Wolfe T. J. (2000). Chronic stress regulates levels of mRNA transcripts encoding β subunits of the GABAA receptor in the rat stress axis. *Brain Res.* 887 118–124. 10.1016/S0006-8993(00)03000-6 11134596

[B13] Dixon M. L. Thiruchselvam R. Todd R. Christoff K. (2017). Emotion and the prefrontal cortex: an integrative review. *Psychol. Bull.* 143 1033–1081. 10.1037/bul0000096 28616997

[B14] Farrant M. Nusser Z. (2005). Variations on an inhibitory theme: phasic and tonic activation of GABAA receptors. *Nat. Rev. Neurosci.* 6 215–229. 10.1038/nrn1625 15738957

[B15] Gilmor M. L. Skelton K. H. Nemeroff C. B. Owens M. J. (2003). The effects of chronic treatment with the mood stabilizers valproic acid and lithium on corticotropin-releasing factor neuronal systems. *J. Pharmacol. Exp. Therap.* 305 434–439. 10.1124/jpet.102.045419 12606697

[B16] Goetz T. Arslan A. Wisden W. Wulff P. (2007). GABA(A) receptors: structure and function in the basal ganglia. *Prog. Brain Res.* 160 21–41. 10.1016/S0079-6123(06)60003-4 17499107 PMC2648504

[B17] Gray C. B. B. Heller Brown J. (2014). CaMKIIdelta subtypes: localization and function. *Front. Pharmacol.* 5:15. 10.3389/fphar.2014.00015 24575042 PMC3920101

[B18] Hammond C. (2015). “Chapter 9 – The ionotropic GABAA receptor,” in *Cellular and Molecular Neurophysiology (Fourth Edition)*, ed. Hammond C. (Boston, MA: Academic Press), 199–219.

[B19] Hayes D. J. Jupp B. Sawiak S. J. Merlo E. Caprioli D. Dalley J. W. (2014). Brain γ-aminobutyric acid: a neglected role in impulsivity. *Eur. J. Neurosci.* 39 1921–1932. 10.1111/ejn.12485 24460847

[B20] Hiser J. Koenigs M. (2018). The multifaceted role of the ventromedial prefrontal cortex in emotion, decision making, social cognition, and psychopathology. *Biol. Psychiatry* 83 638–647. 10.1016/j.biopsych.2017.10.030 29275839 PMC5862740

[B21] Houston C. M. He Q. Smart T. G. (2009). CaMKII phosphorylation of the GABAA receptor: receptor subtype- and synapse-specific modulation. *J. Physiol.* 587 2115–2125. 10.1113/jphysiol.2009.171603 19332484 PMC2697286

[B22] Hsu T. M. Noble E. E. Liu C. M. Cortella A. M. Konanur V. R. Suarez A. N. (2018). A hippocampus to prefrontal cortex neural pathway inhibits food motivation through glucagon-like peptide-1 signaling. *Mol. Psychiatry* 23 1555–1565. 10.1038/mp.2017.91 28461695 PMC5668211

[B23] Lalonde J. Lachance P. E. D. Chaudhuri A. (2004). Monocular enucleation induces nuclear localization of calcium/calmodulin-dependent protein Kinase IV in cortical interneurons of adult monkey area V1. *J. Neurosci.* 24 554–564. 10.1523/jneurosci.1668-03.2004 14724256 PMC6729977

[B24] Lenz B. Röther M. Bouna-Pyrrou P. Mühle C. Tektas O. Y. Kornhuber J. (2019). The androgen model of suicide completion. *Prog. Neurobiol.* 172 84–103. 10.1016/j.pneurobio.2018.06.003 29886148

[B25] Liu R. T. Trout Z. M. Hernandez E. M. Cheek S. M. Gerlus N. (2017). A behavioral and cognitive neuroscience perspective on impulsivity, suicide, and non-suicidal self-injury: meta-analysis and recommendations for future research. *Neurosci. Biobehav. Rev.* 83 440–450. 10.1016/j.neubiorev.2017.09.019 28928071 PMC5730462

[B26] Livak K. J. Schmittgen T. D. (2001). Analysis of relative gene expression data using real-time quantitative PCR and the 2(-Delta Delta C(T)) Method. *Methods* 25 402–408. 10.1006/meth.2001.1262 11846609

[B27] Long P. Mercer A. Begum R. Stephens G. J. Sihra T. S. Jovanovic J. N. (2009). Nerve terminal GABAA receptors activate Ca2+/calmodulin-dependent signaling to inhibit voltage-gated Ca2+ influx and glutamate release. *J. Biol. Chem.* 284 8726–8737. 10.1074/jbc.M805322200 19141616 PMC2659231

[B28] Ludwig B. Roy B. Dwivedi Y. (2019). Role of HPA and the HPG axis interaction in testosterone-mediated learned helpless behavior. *Mol. Neurobiol.* 56 394–405. 10.1007/s12035-018-1085-x 29704202 PMC6204317

[B29] Ma H. Li B. Tsien R. W. (2015). Distinct roles of multiple isoforms of CaMKII in signaling to the nucleus. *Biochim. Biophys. Acta* 1853 1953–1957. 10.1016/j.bbamcr.2015.02.008 25700840 PMC4522395

[B30] Malik Z. A. Stein I. S. Navedo M. F. Hell J. W. (2014). Mission CaMKIIγ: shuttle calmodulin from membrane to nucleus. *Cell* 159 235–237. 10.1016/j.cell.2014.09.023 25303520 PMC7031110

[B31] Melhem N. M. Keilp J. G. Porta G. Oquendo M. A. Burke A. Stanley B. (2016). Blunted HPA axis activity in suicide attempters compared to those at high risk for suicidal behavior. *Neuropsychopharmacology* 41 1447–1456. 10.1038/npp.2015.309 26450815 PMC4832012

[B32] Merali Z. Du L. Hrdina P. Palkovits M. Faludi G. Poulter M. O. (2004). Dysregulation in the suicide brain: mRNA expression of corticotropin-releasing hormone receptors and GABA(A) receptor subunits in frontal cortical brain region. *J. Neurosci.* 24 1478–1485. 10.1523/JNEUROSCI.4734-03.2004 14960621 PMC6730322

[B33] Mick I. Ramos A. C. Myers J. Stokes P. R. Chandrasekera S. Erritzoe D. (2017). Evidence for GABA-A receptor dysregulation in gambling disorder: correlation with impulsivity. *Addict. Biol.* 22 1601–1609. 10.1111/adb.12457 27739164 PMC5697606

[B34] Mott D. (2015). “Chapter 11 – The metabotropic GABAB receptors,” in *Cellular and Molecular Neurophysiology (Fourth Edition)*, ed. Hammond C. (Boston, MA: Academic Press), 245–267.

[B35] Murphy E. R. Fernando A. B. P. Urcelay G. P. Robinson E. S. J. Mar A. C. Theobald D. E. H. (2012). Impulsive behaviour induced by both NMDA receptor antagonism and GABAA receptor activation in rat ventromedial prefrontal cortex. *Psychopharmacology* 219 401–410. 10.1007/s00213-011-2572-1 22101355 PMC3249210

[B36] Petroff O. A. C. (2002). Book review: GABA and glutamate in the human brain. *Neuroscientist* 8 562–573. 10.1177/1073858402238515 12467378

[B37] Poulter M. Du L. Zhurov V. Palkovits M. Faludi G. Merali Z. (2010). Altered organization of GABAA receptor mRNA expression in the depressed suicide brain. *Front. Mol. Neurosci.* 3:3. 10.3389/neuro.02.003.2010 20407580 PMC2854532

[B38] Robbins T. W. Dalley J. W. (2017). “Chapter 7 – Impulsivity, risky choice, and impulse control disorders: animal models,” in *Decision Neuroscience*, eds Dreher J.-C. Tremblay L. (San Diego, CA: Academic Press), 81–93.

[B39] Roy B. Dunbar M. Shelton R. C. Dwivedi Y. (2017). Identification of MicroRNA-124-3p as a putative epigenetic signature of major depressive disorder. *Neuropsychopharmacology* 42 864–875. 10.1038/npp.2016.175 27577603 PMC5312059

[B40] Saberi Moghadam S. Samsami Khodadad F. Khazaeinezhad V. (2019). An algorithmic model of decision making in the human brain. *Basic Clin. Neurosci.* 10 443–449. 10.32598/bcn.9.10.395 32284833 PMC7149951

[B41] Shen K. Teruel M. N. Subramanian K. Meyer T. (1998). CaMKIIbeta functions as an F-actin targeting module that localizes CaMKIIalpha/beta heterooligomers to dendritic spines. *Neuron* 21 593–606. 10.1016/s0896-6273(00)80569-3 9768845

[B42] Sher L. Grunebaum M. F. Burke A. K. Chaudhury S. Mann J. J. Oquendo M. A. (2017). Depressed multiple-suicide attempters—a high-risk phenotype. *Crisis* 38 367–375. 10.1027/0227-5910/a000475 28914095

[B43] Sholler D. J. Merritt C. R. Davis-Reyes B. D. Golovko G. Anastasio N. C. Cunningham K. A. (2020). Inherent motor impulsivity associates with specific gene targets in the rat medial prefrontal cortex. *Neuroscience* 435 161–173. 10.1016/j.neuroscience.2020.03.045 32240784

[B44] Shrivastava A. N. Triller A. Sieghart W. (2011). GABA(A) receptors: post-synaptic co-localization and cross-talk with other receptors. *Front. Cell. Neurosci.* 5:7. 10.3389/fncel.2011.00007 21734865 PMC3123775

[B45] Skelton K. H. Nemeroff C. B. Knight D. L. Owens M. J. (2000). Chronic administration of the triazolobenzodiazepine alprazolam produces opposite effects on corticotropin-releasing factor and urocortin neuronal systems. *J. Neurosci.* 20 1240–1248. 10648728 10.1523/JNEUROSCI.20-03-01240.2000 PMC6774170

[B46] Stefansson J. Chatzittofis A. Nordstrom P. Arver S. Asberg M. Jokinen J. (2016). CSF and plasma testosterone in attempted suicide. *Psychoneuroendocrinology* 74 1–6. 10.1016/j.psyneuen.2016.08.009 27567115

[B47] Steiger J. L. Russek S. J. (2004). GABAA receptors: building the bridge between subunit mRNAs, their promoters, and cognate transcription factors. *Pharmacol. Therap.* 101 259–281. 10.1016/j.pharmthera.2003.12.002 15031002

[B48] Stout S. C. Owens M. J. Lindsey K. P. Knight D. L. Nemeroff C. B. (2001). Effects of sodium valproate on corticotropin-releasing factor systems in rat brain. *Neuropsychopharmacology* 24 624–631. 10.1016/S0893-133X(00)00243-8 11331142

[B49] Svensson A. I. Åkesson P. Engel J. A. Söderpalm B. (2003). Testosterone treatment induces behavioral disinhibition in adult male rats. *Pharmacol. Biochem. Behav.* 75 481–490. 10.1016/S0091-3057(03)00137-0 12873641

[B50] Svensson A. I. Berntsson A. Engel J. A. Söderpalm B. (2000). Disinhibitory behavior and GABAA receptor function in serotonin-depleted adult male rats are reduced by gonadectomy. *Pharmacol. Biochem. Behav.* 67 613–620. 10.1016/S0091-3057(00)00403-2 11164093

[B51] Thiblin I. Runeson B. Rajs J. (1999). Anabolic androgenic steroids and suicide. *Ann. Clin. Psychiatry* 11 223–231. 10.1023/a:1022313529794 10596737

[B52] Thomas K. L. Laroche S. Errington M. L. Bliss T. V. P. Hunt S. P. (1994). Spatial and temporal changes in signal transduction pathways during LTP. *Neuron* 13 737–745. 10.1016/0896-6273(94)90040-X 7917303

[B53] Timberlake M. II Prall K. Roy B. Dwivedi Y. (2018). Unfolded protein response and associated alterations in toll-like receptor expression and interaction in the hippocampus of restraint rats. *Psychoneuroendocrinology* 89 185–193. 10.1016/j.psyneuen.2018.01.017 29414031 PMC5878717

[B54] Trenton A. J. Currier G. W. (2005). Behavioural manifestations of anabolic steroid use. *CNS Drugs* 19 571–595. 10.2165/00023210-200519070-00002 15984895

[B55] Ucha M. Roura-Martinez D. Contreras A. Pinto-Rivero S. Orihuel J. Ambrosio E. (2019). Impulsive action and impulsive choice are differentially associated with gene expression variations of the GABAA Receptor Alfa 1 Subunit and the CB1 receptor in the lateral and medial orbitofrontal cortices. *Front. Behav. Neurosci.* 13:22. 10.3389/fnbeh.2019.00022 30842730 PMC6391359

[B56] Wood R. I. Armstrong A. Fridkin V. Shah V. Najafi A. Jakowec M. (2013). ‘Roid rage in rats? Testosterone effects on aggressive motivation, impulsivity and tyrosine hydroxylase. *Physiol. Behav.* 11 6–12. 10.1016/j.physbeh.2012.12.005 23266798 PMC3615053

[B57] Wu C. Sun D. (2015). GABA receptors in brain development, function, and injury. *Metab. Brain Dis.* 30 367–379. 10.1007/s11011-014-9560-1 24820774 PMC4231020

[B58] Wu Y. Shen B. Liao J. Li Y. Zilioli S. Li H. (2019). Single dose testosterone administration increases impulsivity in the intertemporal choice task among healthy males. *Horm. Behav.* 118:104634. 10.1016/j.yhbeh.2019.104634 31765657

[B59] Yan X. X. Baram T. Z. Gerth A. Schultz L. Ribak C. E. (1998). Co-localization of corticotropin-releasing hormone with glutamate decarboxylase and calcium-binding proteins in infant rat neocortical interneurons. *Exp. Brain Res.* 123 334–340. 10.1007/s002210050576 9860272 PMC3786772

[B60] Yin H. Pantazatos S. P. Galfalvy H. Huang Y.-Y. Rosoklija G. B. Dwork A. J. (2016). A pilot integrative genomics study of GABA and glutamate neurotransmitter systems in suicide, suicidal behavior, and major depressive disorder. *Am. J. Med. Genet. Part B Neuropsychiatr. Genet.* 171 414–426. 10.1002/ajmg.b.32423 26892569 PMC4851346

